# Solar-Driven Thin Air Gap Membrane Distillation with a Slippery Condensing Surface

**DOI:** 10.1021/acs.est.4c06470

**Published:** 2024-11-16

**Authors:** Hongxia Li, Aikifa Raza, Noora Ali AlMarzooqi, Meera AlMehrzi, Alaa Shaheen, Faisal AlMarzooqi, TieJun Zhang

**Affiliations:** † Department of Mechanical and Nuclear Engineering, Khalifa University of Science and Technology, Abu Dhabi 127788, United Arab Emirates; ‡ Technology Innovation Institute, Abu Dhabi 9639, United Arab Emirates; § Department of Chemical and Petroleum Engineering, Khalifa University of Science and Technology, Abu Dhabi 127788, United Arab Emirates; ∥ Center for Membranes and Advanced Water Technology (CMAT), Khalifa University of Science and Technology, Abu Dhabi 127788, United Arab Emirates

**Keywords:** membrane distillation, dropwise condensation, slippery surface, permeate flooding, solar desalination

## Abstract

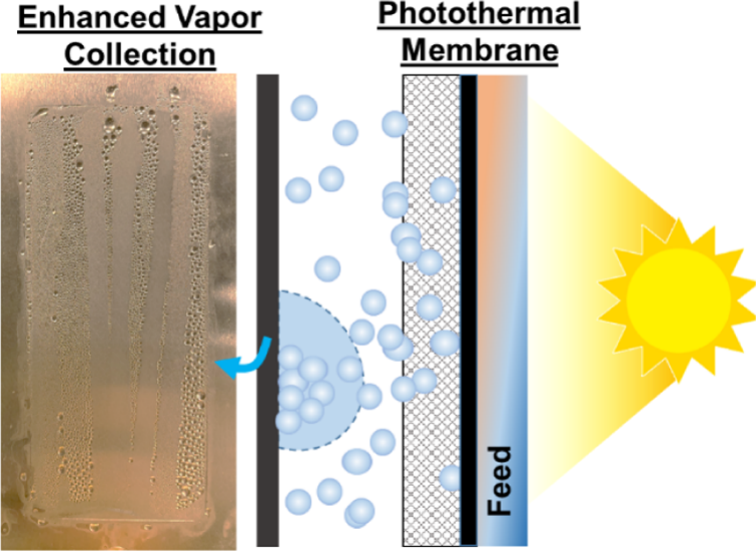

Membrane-based desalination is essential for mitigating global water scarcity; yet, the process is energy-intensive and heavily reliant on fossil fuels, resulting in substantial carbon emissions. To address the challenges of treating seawater, produced water, brackish groundwater, and wastewater, we have developed a thin air gap membrane distillation (AGMD) system featuring a novel slippery condensing surface. The quasi-liquid slippery surface facilitates efficient condensate water droplet removal, allowing for the implementation of a 1 mm thin air gap. This advancement has led to a 2-fold increase in permeate flux without lowering the thermal efficiency while preventing permeate flooding. Furthermore, the thin AGMD system, employing a cost-effective zirconium nitride/poly(vinylidene fluoride) (ZrN-PVDF) composite membrane, has been demonstrated for solar-driven desalination. Experimental results indicate that reducing the air gap from 2 to 1 mm enhances the permeate flux by 150%.

## Abstract 2

A solar-driven thin air gap membrane distillation system has been developed to boost clean water production by 150%, promising great potential in high-salinity and unconventional water purification.

## 1 Introduction

Watering humanity puts enormous environmental pressure, particularly in the Middle East and North Africa, the Southwestern USA, and Northwestern China. Various desalination and water purification systems have been widely deployed to increase the freshwater supply around the world. Different from traditional thermal desalination, membrane desalination with reverse osmosis (RO) is gaining more popularity, though RO desalination often suffers from high energy consumption with significant CO_2_ emissions and chemical waste discharge.^1,2^ To date, globally installed desalination capacities are contributing 76 million tons of CO_2_ per year,^3^ in particular, RO-based membrane desalination imposes a more significant carbon footprint per m^3^ as the energy consumption of the RO membrane increases dramatically with the salinity of seawater. Utilizing clean energy is the most effective way to mitigate the energy and environmental-associated impacts of membrane desalination technologies.^4,5^ Complementary to photovoltaic-powered RO, solar-driven membrane distillation (MD)^6^ offers another promising solution as it directly uses solar thermal energy, and it can also deal with hyper-saline water, such as brine (including that from RO) and produced water from underground. In particular, near-perfect solar-to-heat conversion has been achieved by novel multifunctional nanocomposite membranes^7−14^ with high solar absorptivity for the MD process. With a significant reduction of energy consumption and carbon emission in the photothermal and hybrid systems,^15,16^ solar-driven MD has demonstrated great potential, especially in the arid regions endowed with copious solar resources.

Membrane distillation (MD) technologies, including water/permeate gap membrane distillation (WGMD or PGMD) and air gap membrane distillation (AGMD),^17−20^ have garnered significant attention due to their unique advantages and different applicability across various fields. WGMD generally achieves higher permeate flux than AGMD, ranging between 90 and 140%, but with a sacrifice of thermal efficiency due to severe heat conduction loss.^18^ Membrane wetting is also a significant concern in WGMD when dealing with volatile substances or feedwater that contains surfactants or organic compounds. On the opposite, AGMD is particularly valued for its high thermal efficiency,^19,20^ insensitive to volatile substances with a low surface tension, and thus capable of harvesting purified water from a wide range of unconventional sources, such as produced water, brackish groundwater, and wastewater. When driven by solar energy with a constant energy flux of around 1000 W m^–2^, the air gap in AGMD is of great importance in minimizing the conduction heat loss from the photothermal membrane to the condenser.

**Figure 1:**
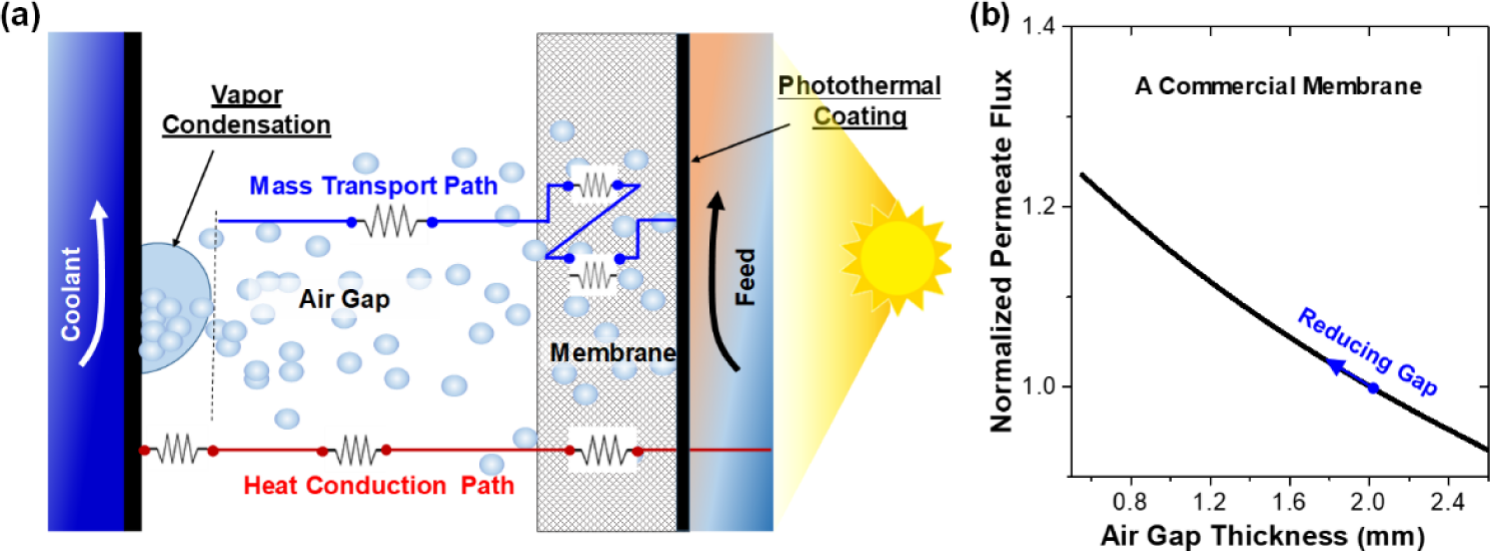
Heat and mass transport across the air gap and the role of vapor condensation in the solar-driven AGMD process. (a) Illustration of the heat and mass transport across the air gap; (b) sensitivity analysis of permeate flux to air gap thickness.

However, AGMD tends to produce lower permeate flux and is sensitive to air gap thickness.^18,21^ Based on the transport process in AGMD illustrated in Figure 1a, we have evaluated the sensitivity of permeate flux to the air gap thickness. Figure 1b demonstrates how the water flux increases as the air gap thickness decreases in a commercial membrane. Detailed calculations can be found in the Supporting Information. To minimize the air gap, tremendous research efforts have been dedicated to rapid condensate removal by designing novel condensing surfaces.^22−24^ In AGMD, a good condensing surface is beneficial from two aspects: (i) reducing the condensation heat transfer resistance, which leads to a low vapor pressure at the cooling side to drive the vapor transport across the air gap, and (ii) avoiding the flooding of the air gap, thus potentially reducing the mass transport resistance by narrowing the air gap. Note that vapor condensation and collection is a crucial process not only in AGMD^20,25^ but also in many emerging clean water production applications, including atmospheric water harvesting^26−28^ and solar thermal wastewater treatment.^12,29,30^ Given this context, we are motivated by a recent breakthrough in slippery surface design for condensate removal, especially for small-sized droplet removal for broad water applications.

Recently, a new type of quasi-liquid, also called all-solid, slippery surface has gained significant attention.^31−33^ This kind of quasi-liquid slippery surface is created by covalently tethering one end of a long-chain polymer on solid substrates while keeping the other end mobile, which is different from conventional slippery surfaces such as microstructured hydrophobic surfaces and lubricant-infused surfaces.^34−38^ The grafted flexible brush-like polymer of the quasi-liquid surface is able to improve the slippage level of the substrate for rapid condensate removal, while the slippage of conventional slippery surfaces would be lost due to damage of texture or depletion of lubricant via drainage or repeated use.^39−41^ Another concern of employing lubricant-infused surfaces for the AGMD process is that the collected freshwater is easily contaminated by the lubricant,^40^ causing additional environmental problems. Fortunately, these concerns related to texture damage and lubricant depletion are greatly mitigated once quasi-liquid slippery surfaces are applied in AGMD. Accordingly, the durability and contamination issues of conventional slippery surfaces can be resolved for broader freshwater production applications.

In the present work, we propose to develop an advanced slippery condensing surface for efficient water collection and scalable composite membranes for broadband sunlight absorption toward high-performance solar-driven AGMD. For condensation enhancement, two different slippery surfaces, including a microstructured hydrophobic surface and a quasi-liquid slippery surface, are employed by functionalizing the commercially used condensing surface. A series of comparative experiments are conducted to evaluate the performance of slippery surfaces in enhancing permeate flux and the gain output ratio (GOR). These experiments are carried out under controlled conditions, with varying coolant temperatures set at 5, 10, 15, and 20 °C. The underlying mechanisms are further investigated by characterizing surface wettability and measuring the adhesion forces using atomic force microscopy. Additionally, we perform a statistical analysis of the condensate droplet size distributions on the three different surfaces following the AGMD process. The benefits of using slippery condensing surfaces are systematically evaluated in AGMD with a thin air gap. Another contribution of this work is solar-driven AGMD with zirconium nitride (ZrN) PVDF composite membranes, which exhibit superior light absorption and vapor permeability simultaneously.

## 2 Experimental Materials and Procedures

### 2.1 Slippery Surface Fabrication

**Figure 2:**
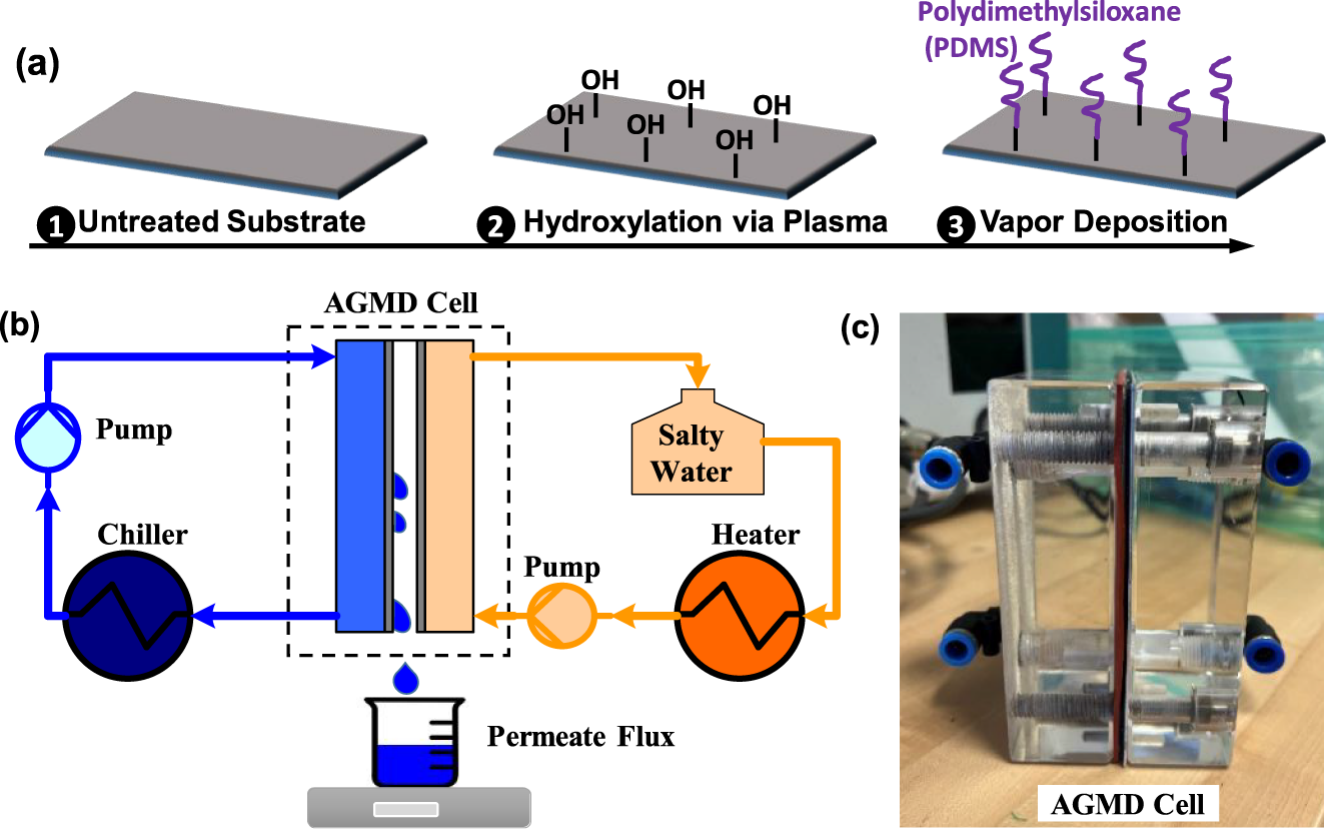
Quasi-liquid slippery surface as a condensing surface in the AGMD system. (a) Fabrication process of the quasi-liquid slippery surface by covalently tethering chlorine-terminated polydimethylsiloxane (PDMS). (b) Schematic diagram of the AGMD experimental setup. (c) Photo of the flat cell membrane module used in the setup.

To get the microstructured hydrophobic surface, bare aluminum was chemically etched to create surface roughness and then silanized for ensuring hydrophobicity (detailed approach given in our previous publication^42^). The quasi-liquid slippery surface was introduced to aluminum through vapor deposition (see Figure 2a) by tethering chlorine-terminated polydimethylsiloxane (PDMS),^31,43^ which has a molecular weight of *M*_W_ 4000. In particular, the smooth bare aluminum plate was treated with oxygen plasma to create a hydroxyl group. Then, it was placed upside down on a 15 cm-diameter glass Petri dish, which was filled with 200 μL of liquid chlorine-terminated PDMS oligomer. Both the aluminum plate and Petri dish were placed inside a desiccator in connection with a vacuum pump for 2 h. Finally, the samples were rinsed in a water bath inside the shaker for 10 min to ensure uniform rinsing and clean untethered PDMS residues. The samples were ready for use after they were dried with nitrogen gas.

### 2.2 Surface Wettability Characterization

Wettability is essential for surfaces to ensure effective vapor condensation. Static and dynamic contact angles on the as-fabricated surfaces were measured by a goniometer (DM-501, Kyowa Interface Science Co., Ltd.). The microscale surface morphologies were characterized with a scanning electron microscope (SEM, FEI Quanta 250). An atomic force microscope (AFM, Peak Force QNM, Bruker) was used to map the adhesion forces between a hydrophilic probe and condensing surfaces. Monolithic silicon AFM probes (Multi75-G, Ted Pella, Inc.) were used for force modulation. The AFM cantilevers used here have a resonant frequency of 75 kHz, a force constant of 3 N/m, and a tip with a radius of 10 nm. The spring constants and deflection sensitivities of the probes used for both adhesion mapping and topography imaging were calibrated at the start of each experiment with the thermal tune method^44^ by measuring their contact against a clean mica surface. Before each experiment, the probes were thoroughly rinsed with ethanol to remove any physically adsorbed substances, followed by drying with nitrogen gas. This procedure is critical to restore the intrinsic hydrophilicity of the silicon oxide-covered AFM probe.

### 2.3 Conventional and Solar-Driven AGMD Setups

A flat sheet AGMD system is employed to characterize the permeate flux and gain output ratio or thermal efficiency (see Figure 2b). The main components of the setup are the feedwater loop, coolant loop, and flat sheet membrane module, as shown in Figure 2c. The dimension of the cooling plate is 12.7 cm × 10 cm with four assembling holes. The effective condensing surface is 8 cm × 4 cm at the center, which is the same as the membrane surface area. The functionalized condensing surfaces were assembled in the flat sheet membrane cell for performance evaluation, and the simulated seawater with 3.5 wt % NaCl was used as feedwater. To facilitate the characterization of various condensing surfaces, the temperature of the hot feedwater was fixed at 50 °C, and the volume flow rate was 200 mL/min. By changing the coolant temperature ranging from 5 to 20 °C, we were able to investigate the vapor condensation performance under different subcooling conditions, and the condensate water was collected and weighed in real-time. In conventional thermal AGMD systems, commercial PVDF membranes (pore size of 0.45 μm, Thermo Scientific) were used, while in solar-driven AGMD, a solar simulator and light-absorbing membrane were used to provide the energy for heating up the feedwater. The light-absorbing membrane was made of ZrN-PVDF and fabricated with the nonsolvent induced phase-inversion (NIPS) method.^45^ The absorptivity/reflectivity of composite ZrN-PVDF membranes was recorded in the wavelength range 250–2000 nm using a UV–vis–NIR spectrophotometer (LAMBDA 1050). Experimental characterization was performed for conventional thermal AGMD and solar-driven AGMD with functionalized condensing surfaces and ZrN-PVDF light-absorbing membranes for a systematic performance assessment. In our solar AGMD experiments, the room-temperature simulated seawater with 3.5 wt % NaCl was also used as feedwater without preheating, and the coolant temperature was set at 10 °C.

## 3 Results and Discussion

### 3.1 Condensing Surface Morphology and Wettability Conditions

**Figure 3:**
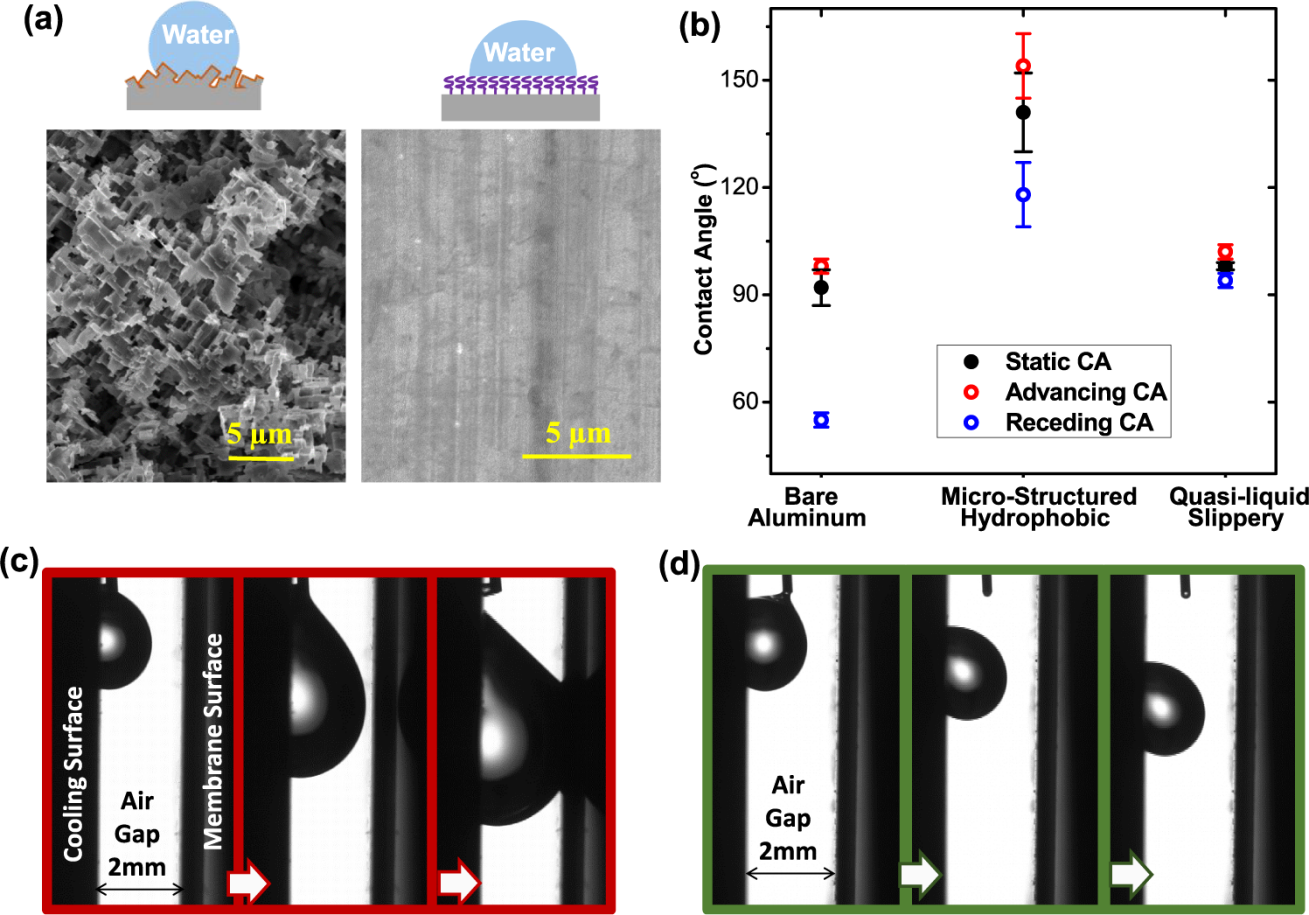
Surface morphologies and wettability characterization of the functionalized condensing surfaces. (a) SEM images of the microstructured hydrophobic surface and quasi-liquid slippery surface for vapor condensation. (b) Surface wettability and contact angle hysteresis characterization for bare aluminum surface, microstructured hydrophobic surface, and quasi-liquid nontextured surface. (c,d) Droplet sliding tests in an air gap with 2 mm width for the commercial bare aluminum surface (c) and the quasi-liquid slippery surface (d).

In this work, we evaluated three different condensing surfaces, including a commercially used bare aluminum surface, a microstructured hydrophobic surface, and a quasi-liquid slippery surface. The surface morphologies of the as-modified slippery condensing surfaces are presented in Figure 3a. As seen from the SEM images, the hydrophobic surface has microscale structures from chemical etching, while the quasi-liquid slippery surface does not show any microscale surface textures. As for surface wettability, the static contact angle (CA) and contact angle hysteresis (CAH) of the three condensing surfaces are shown in Figure 3b. The commercial bare aluminum surface has a static CA of around 92° and a relatively high CAH with an advancing CA of 98° and a receding CA of 55°. After surface texturing and silanization, the surface exhibits good hydrophobicity with a static CA of 141°, but the CAH is not noticeably improved. On the contrary, the quasi-liquid slippery surface has a static CA similar to that of the bare aluminum surface but with a considerably lower CAH, where the advancing CA is around 102° and the receding CA is 94°. As expected, the ultralow CAH results from the covalently grafted brush-like long molecular chains of PDMS. We also checked the droplet sliding capability in a mimicked AGMD process, where a 2 mm gap is maintained between the slippery surface and the membrane. As shown in Figure 3c, the droplet got stuck on the bare aluminum surface. When its size increases, the droplet touched the membrane and formed a water bridge in the air gap, causing performance degradation of traditional AGMD as revealed in Figure 1. However, on the quasi-liquid slippery surface, the droplet is able to slide away easily without touching the membrane (see Figure 3d).

**Figure 4:**
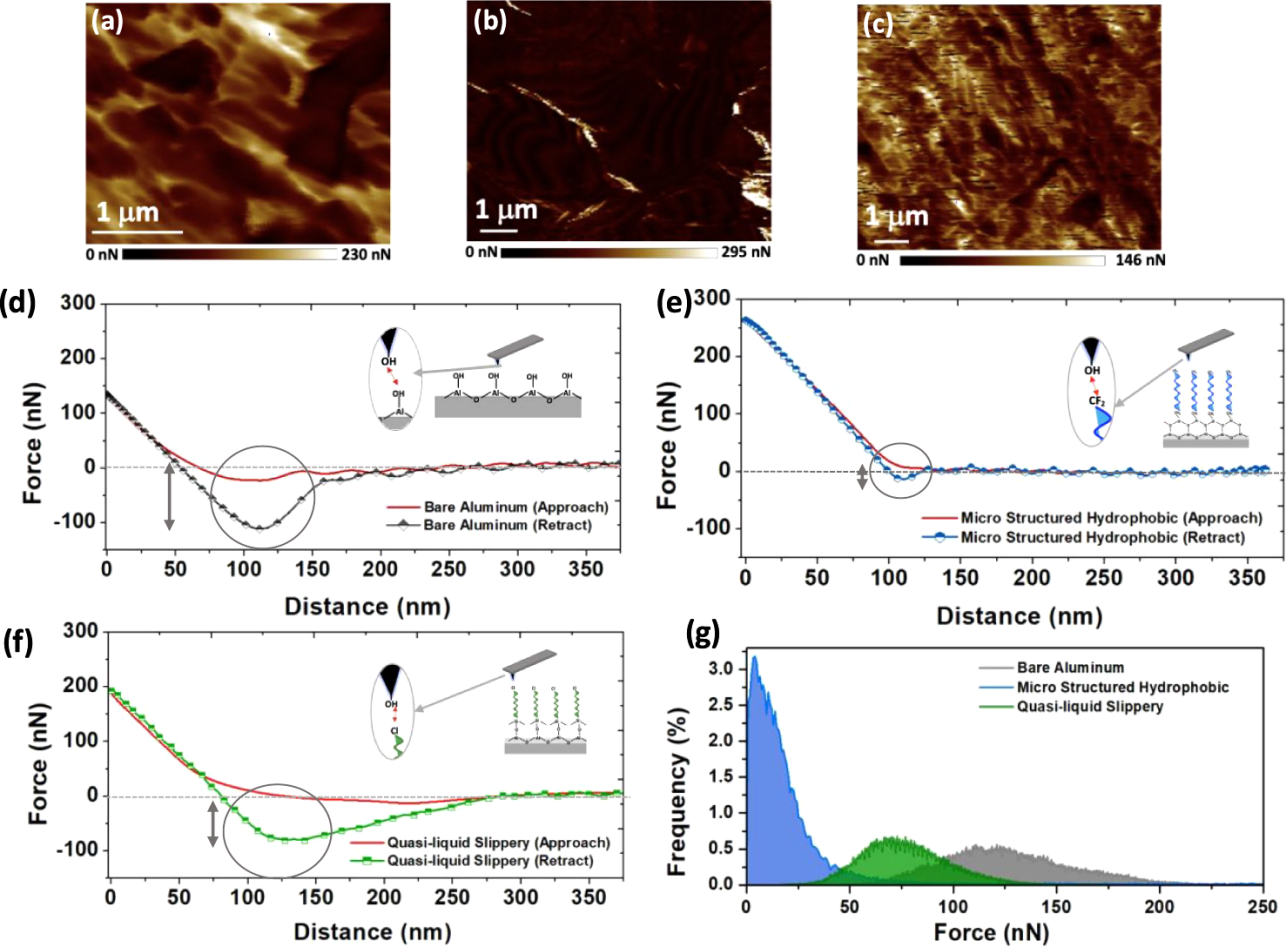
Adhesion maps of three different surfaces: (a) bare aluminum, (b) microstructured hydrophobic, and (c) quasi-liquid slippery. Selected approach and retract curves show the interaction behavior of the probe with (d) bare aluminum, (e) microstructured hydrophobic, and (f) quasi-liquid slippery surfaces, respectively. Insets in parts (d,f) show schematics of probe-condensing surface interactions at the molecular level. (g) Histogram of measured adhesion values from parts (a–c). The reported adhesion forces are measured by taking the absolute minimum value of retract force curves at various points of the adhesion maps shown in parts (a–c).

To explain the water droplet mobility on various condensing surfaces, we also performed force–distance (FD) spectroscopy with an atomic force microscope to investigate the intrinsic adhesion forces. Figure 4a–c shows adhesion maps of bare aluminum, microstructured hydrophobic, and quasi-liquid slippery surfaces. At each location on the map, the adhesion force is obtained from the FD measurement with the hydrophilic probe when it approaches and retracts from the sample surface. In Figure 4d, adhesion occurs when the probe approaches the bare aluminum surface (see the approach curve), owing to the van der Waals forces of attraction and hydrogen bonding among the probe and sample surface. As the probe retracts, the adhesion force causes a deflection in the cantilever, and the adhesion force is calculated from the cantilever deflection (see the retract curve). The representative FD curves for bare aluminum, microstructured hydrophobic, and quasi-liquid slippery surfaces are shown in Figure 4d–f, respectively. Figure 4g also summarizes the histogram comparing the distribution of measured adhesion, which was generated with the absolute minimum values from each retracted part of the FD curve for three different surfaces. In short, the bare aluminum surface shows the highest adhesion toward the hydrophilic probe with an average adhesion of 140 nN, while adhesion forces for microstructured hydrophobic and quasi-liquid slippery surfaces are in the range of 10–30 and 50–100 nN, respectively. Since a contact angle is determined by the balance of surface adhesion and cohesion forces, a low contact angle indicates relatively high surface adhesion. The FD spectroscopy results are aligned with the intrinsic contact angle, as measured in Figure 3b.

### 3.2 AGMD Performance and Gain Output Ratio

**Figure 5:**
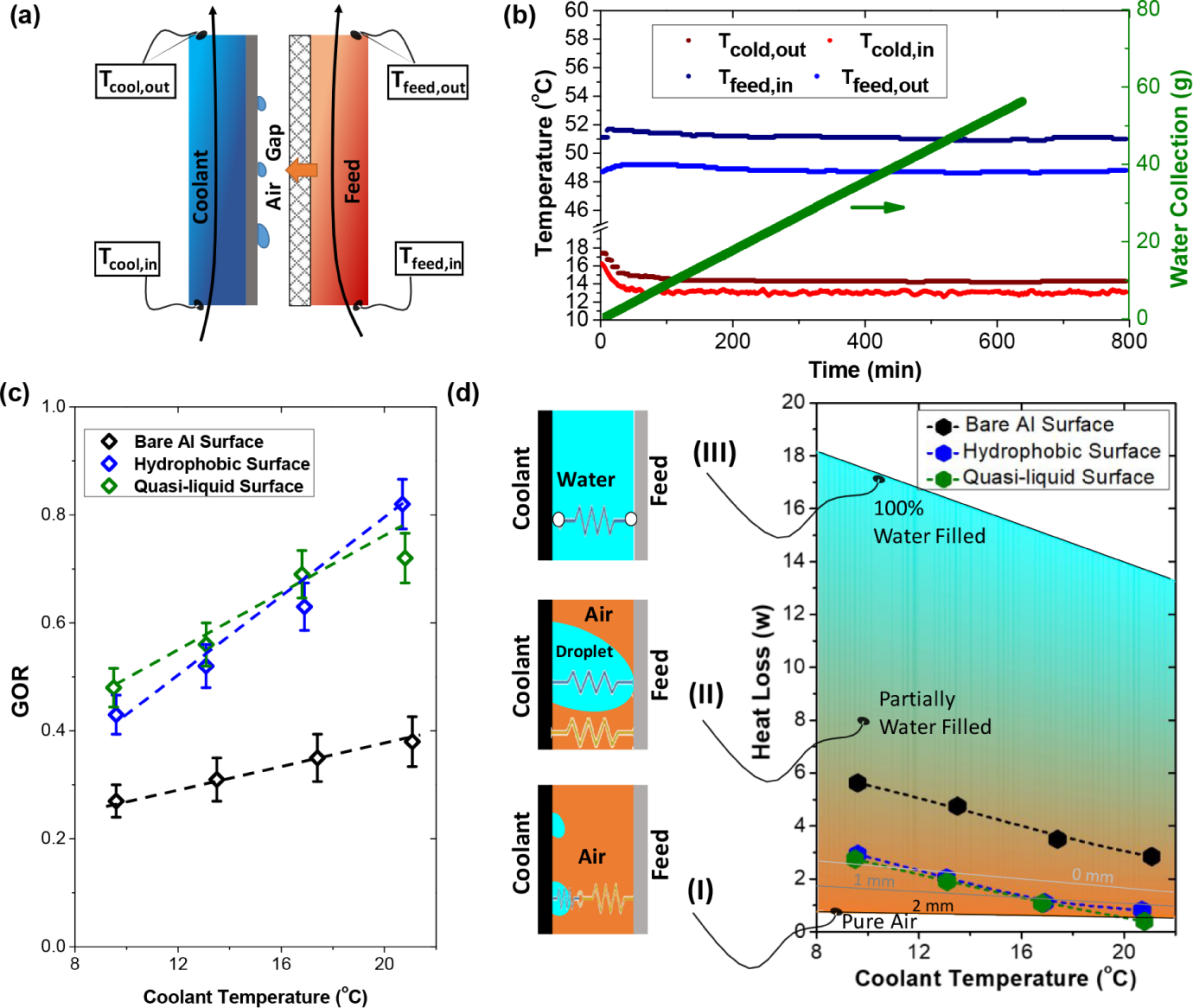
Gain output ratios and heat conduction losses of AGMD with a 2 mm air gap. (a) Schematic of the measurements during the AGMD process. (b) Temperature and permeate flux of the microstructured hydrophobic surface with a feed temperature of 50 °C and a coolant temperature of 10 °C. (c) GORs of three different condensate surfaces. (d) Heat loss due to water filling in the air gap.

The gain output ratio (GOR) is a crucial parameter representing thermal efficiency and indicating how much incoming energy is retained for freshwater production in the AGMD process. It is calculated as the ratio of the latent heat of evaporation of a unit mass of permeate water to the amount of energy used by a desalination system to produce that unit mass of permeate.^19,20,46^ For conventional AGMD, GOR is the ratio of the evaporation enthalpy to the heat energy input from the feedwater in eq 1
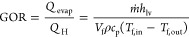
1where *Q*_evap_ is the evaporation enthalpy of water, and it is calculated as the product of permeate flux  and the latent heat of vaporization *h*_lv_. The total energy input is the sum of evaporation enthalpy, conduction loss from the air gap, and other thermal losses. It can be obtained from the measured inlet temperature *T*_f,in_, outlet temperature *T*_f,out_, and the volume flow rate *V*_f_ of the feed side (see Figure 5a). Note that in the experiments, the permeate flux  is measured in real-time. For the GOR of conventional AGMD, the heat or thermal energy represents the enthalpy change of the feedwater through the measurement of the inlet and outlet temperatures. The volume flow rate of the feed is fixed at 200 mL/min.

Similarly, for solar-driven AGMD, the GOR is calculated as the ratio of the evaporation enthalpy to the solar energy input in eq 2.
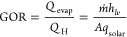
2where *A* is the membrane surface area under solar irradiance, *q*_solar_ is the one-sun solar irradiance (1000 W m^–2^) during the experiments.

As shown in Figure 5, the GOR is experimentally evaluated for three different condensing surfaces under a wide range of coolant temperatures from 10 to 25 °C. During the experiments, the permeate flux is measured in real-time, as are the inlet and outlet temperatures for both the feed and coolant sides. All these measurements were plotted in Figure 5b for the case of a microstructured hydrophobic condensing surface at a coolant temperature of 10 °C. The permeate flux is 1.32 kg h^–1^ m^–2^, as calculated from the slope of the green curve in Figure 5b. Here, we compare the performance of solar AGMD with three different condensing surfaces. As presented in Figure 5c, the GOR of the microstructured hydrophobic surface and the quasi-liquid surface are much higher, almost two times that of the one with the commercially used aluminum surface. For instance, when operating with the highest coolant temperature of 20 °C, the GOR of the aluminum condensing surface is only 0.39, while the efficiency of the quasi-liquid surface is as high as 0.82, and that of the microstructured hydrophobic one is 0.73. The quasi-liquid slippery surface exhibits a comparable GOR with the microstructured surface but is slightly better when the coolant temperature increases.

High GOR means low heat conduction loss through the air gap, essentially a reflection of the water filling status inside the air gap: no water bridge, partially filled, or completely flooded, as shown in Figure 5d. We also mapped the heat conduction loss when the gap is partially filled with permeate water, and the orange-to-blue color transition in Figure 5d indicates higher heat loss. In an ideal AGMD, the condensate droplets are shed effectively, and the heat conduction loss is mitigated owing to the low conductivity of air (Case I). If a water droplet is stuck on the condensing surface, it would keep growing and eventually bridge the membrane and condensate surface, as illustrated in Case II. Correspondingly, a shortcut of heat conduction is created across the gap. The increased number of these bridging droplets results in increased heat loss. The worst case is that the gap is completely flooded with condensate water (see Case III), which would suffer from the largest heat loss. On the map of Figure 5d, we also plotted the heat conduction loss during the AGMD process for three different cooling surfaces. For all the condensing surfaces, the water filling level becomes more severe when the coolant temperature is lower. For the 2 mm air gap used here, both the microstructured hydrophobic surface and the quasi-liquid slippery surface are able to shed the condensate effectively to avoid significant heat loss, ultimately exhibiting higher GOR in comparison with the commercial aluminum condensing surface.

### 3.3 Thin AGMD Enabled by Rapid Condensate Removal

**Figure 6:**
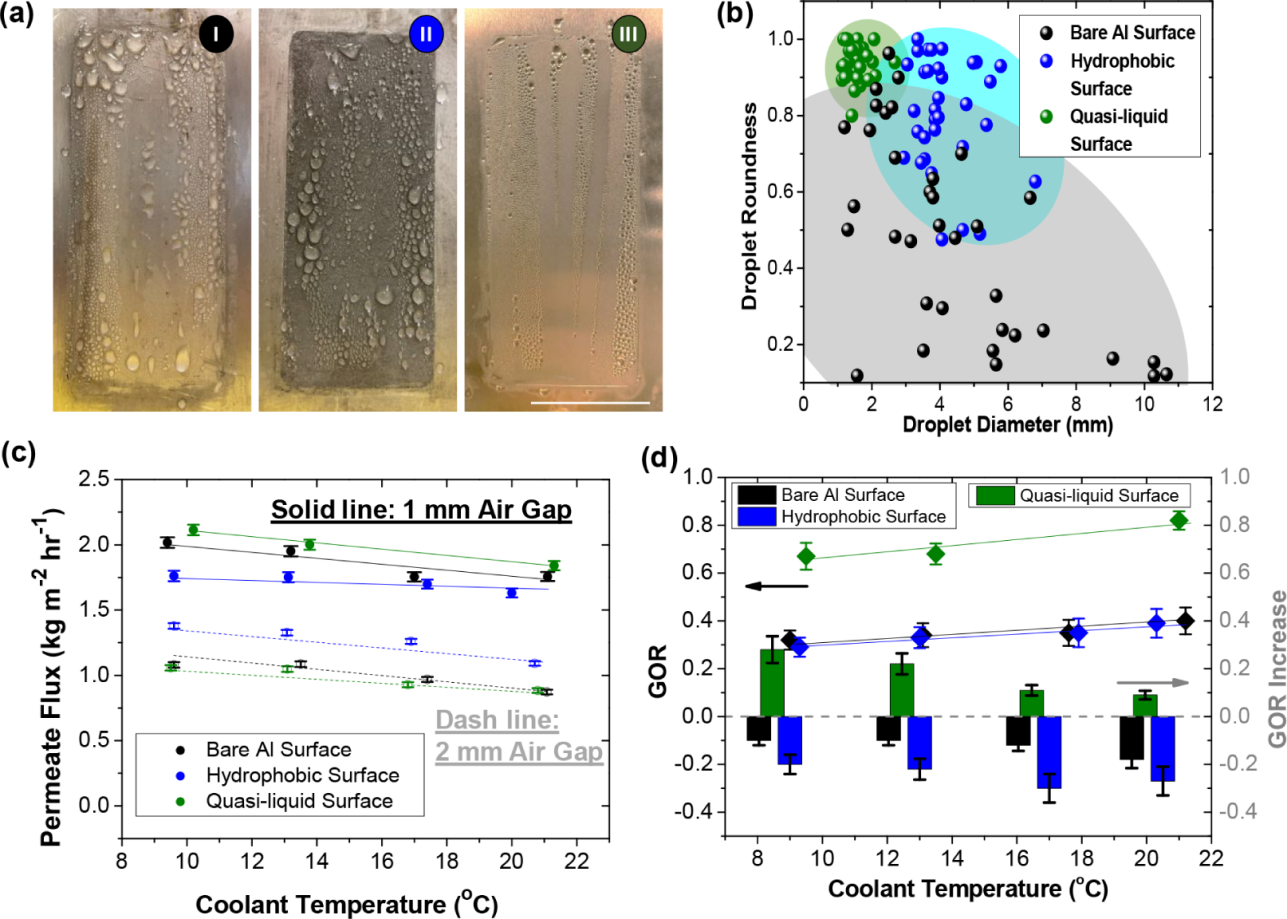
Thin AGMD enabled by rapid condensate removal. (a) Droplet morphologies on the condensing surface after 12 h of operation. (I. aluminum surface, II. nanostructured hydrophobic surface, III. nontextured quasi-liquid slippery surface). (b) Droplet size distribution on the condensate surface of the AGMD cell. (c) Permeate flux enhancement and (d) change of GOR when reducing the air gap width from 2 to 1 mm. A quasi-liquid slippery surface is able to maintain the high GOR for thin AGMD with a 1 mm air gap.

GOR and water filling in the air gap highly depend on the capability of the condensing surface in condensate droplet removal. Therefore, we examined the droplet morphology on three different condensate surfaces after 12 h of AGMD operation (see Figure 6a). A few millimeter-sized droplets are visible on both the aluminum surface and the microstructured hydrophobic surface, while such droplets do not exist on the quasi-liquid slippery surface. We also statistically analyzed the droplet size distribution and roundness for these three condensing surfaces. The image is processed and analyzed by using ImageJ^47^ to obtain the droplet radii. The roundness is calculated as the ratio of the radii of inscribed and circumscribed circles of the droplet outline. The results are presented in Figure 6b. The droplets on the quasi-liquid surface are compactly clustered in the green shadowed area, with all droplet sizes below 2 mm and roundness above 0.8. Another extreme is the commercial aluminum surface, where the droplets are scattered in the gray area with a broad size range from 1 to 10 mm. The microstructured hydrophobic surface has the intermediate distribution among these three condensing surfaces. When the air gap width is only 2 mm in the AGMD experiments, those big droplets would bridge the membrane and cooling surface, which is the root cause of the low GOR. Moreover, the roundness of the droplet shape is also an important indicator of the ease of contact line movement. The observations in Figure 6b are also aligned with our previous CAH. The nontextured quasi-liquid surface has the smallest CAH and the highest roundness.

The easy droplet shedding, especially for small droplets, can empower thin AGMD without causing flooding. In other words, it can decrease the vapor transport resistance for permeate flux enhancement without significant heat loss. Hence, we reduced the gap width from 2 to 1 mm, with results presented in Figure 6c,d. For all the condensing surfaces, the fluxes have significant improvements, almost doubled regardless of the coolant temperature. That is because of the smaller vapor diffusion resistance in the thinner air gap. However, the changes in GOR deviate considerably among the three different condensing surfaces. When reducing the air gap from 2 to 1 mm, the hydrophobic surface and the bare aluminum surface exhibit the same GOR, around 50% lower than the quasi-liquid surface. Compared with the 2 mm AGMD configuration, only the quasi-liquid surface can maintain its high GOR with a slight increase. On the opposite, the microstructured hydrophobic surface shows the highest GOR decrease after narrowing the gap to 1 mm. For instance, in the case of the coolant temperature of 20 °C, the GOR drops by around 0.29 (see Figure 6d). From the previously mentioned droplet size distributions, it is not difficult to understand that the severe heat loss is because the large condensate droplets bridge the membrane and cooling surface when the gap is reduced. As a result, the advantage of using a microstructured hydrophobic surface does not exist anymore when reducing the air gap width in comparison with the commercial aluminum surface. In fact, the quasi-liquid slippery surface can render thin AGMD achieving even higher GOR owing to its low CAH and extraordinary droplet shedding capability.

### 3.4 High-Performance Solar-Driven AGMD with Thin Air Gap

**Figure 7:**
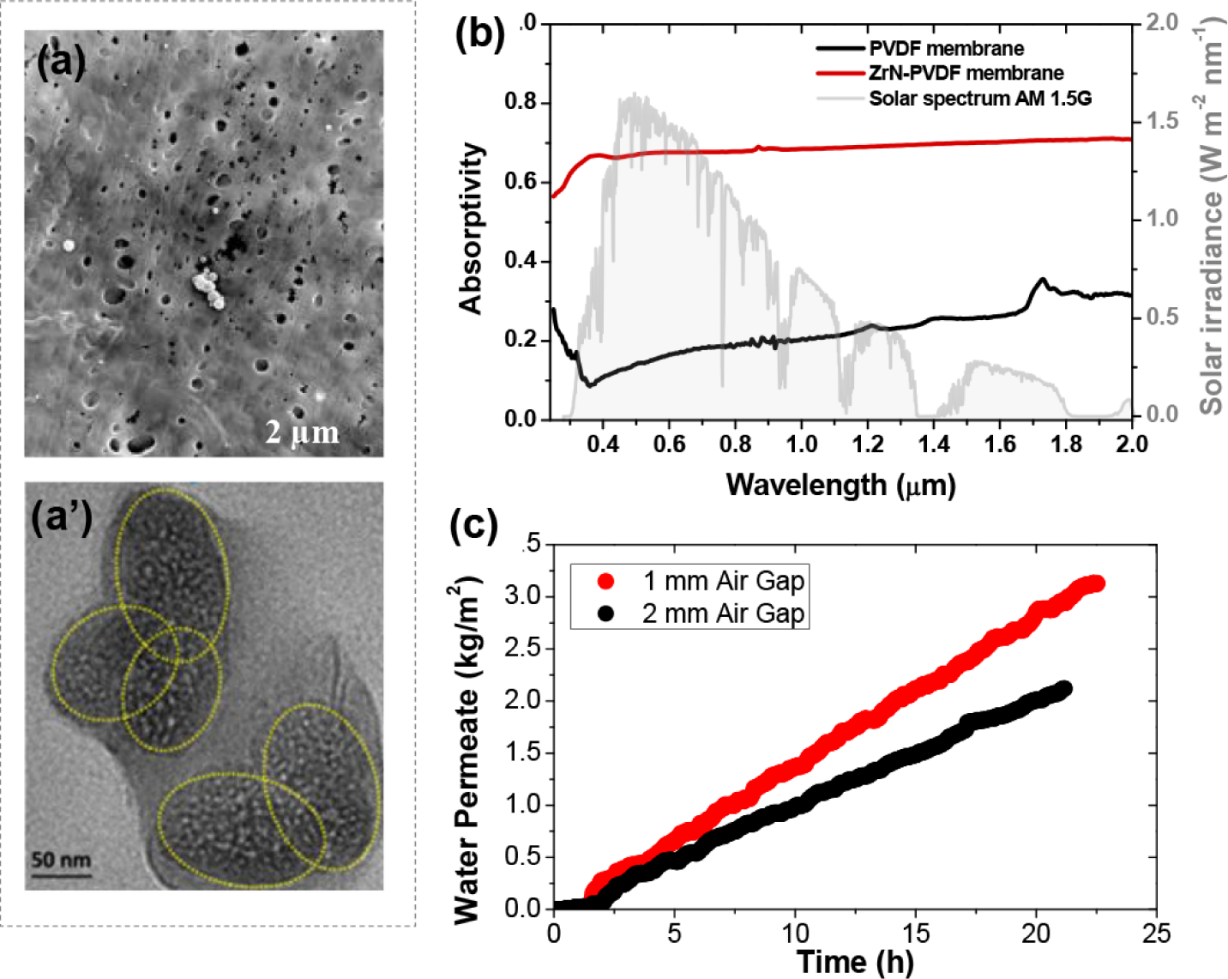
(a) High-resolution SEM image of the ZrN-PVDF composite membrane and (a’) TEM image of ZrN nanoparticles. (b) Measured absorptivity of pure PVDF and 7 wt % ZrN-PVDF composite membrane in the wavelength range of 250–2000 nm. (c) Permeate flux enhancement under one-sun illumination when narrowing the gap width from 2 to 1 mm.

The quasi-liquid slippery surface can also be applied to enhance condensation in solar-driven thin AGMD. Unlike conventional thermal AGMD, solar energy is harvested by a light-absorbing ZrN-PVDF membrane to heat the feedwater. Here, we have utilized ZrN nanoparticle composite membranes toward high-performance AGMD from our recent publication.^45^ SEM image in Figure 7a shows the top surface morphology of the ZrN-PVDF composite membrane.Transmission electron microscope (TEM) image in Figure 7a’ shows various elliptical-shaped ZrN nanoparticles. The addition of 7 wt % ZrN nanoparticles to the membranes helps achieve broadband sunlight absorptance and improve solar absorptance by approximately 40% in the range between 400 and 1800 nm in comparison with the pristine PVDF membrane, as presented in Figure 7b. The averaged solar absorptivity of the ZrN/PVDF solar-absorbing membrane is around 70% across the wavelength range of 250–2000 nm. The solar-driven AGMD experiments are conducted under one-sun illumination, and the permeate flux is monitored in real-time as plotted in Figure 7c. The permeate flux is 0.145 kg m^–2^ h^–1^ for the 1 mm air gap, while the flux is only 0.099 kg m^–2^ h^–1^ for the 2 mm air gap. A 150% flux enhancement is achieved for solar-driven thin AGMD. In the Supporting Information, we compared the gain output ratio (GOR) and permeate flux of our thin air gap solar-driven AGMD with the previously reported data. With our low-cost solar-absorbing membrane (∼60% absorptivity only), the 2 mm air gap configuration exhibits an intermediate performance among all of the reported solar-driven MD tests (see Table S2).

The solar-driven distillation system is demonstrated as a sustainable approach for water production. Still, it exhibits a lower GOR compared to conventional thermal desalination methods, primarily due to constraints in heat recovery and the low energy density of solar energy (∼1000 W m^–2^ only). However, there are several avenues for potential enhancement that could help mitigate this disparity. Notable strategies include the implementation of multistage MD configurations to maximize heat utilization,^48^ advanced heat recovery mechanisms, and optimized integration with solar thermal systems, such as concentrated solar power and thermal energy storage. Additionally, advancements in membrane materials, characterized by higher thermal conductivity and permeability, along with innovative system designs, such as vacuum-assisted and air gap MD, present promising opportunities to enhance the overall GOR. Together with the proposed thin air gap MD, these improvements could establish solar-driven MD as a more sustainable and economically viable option for desalination in off-grid and resource-constrained environments.

## Supporting Information Available

Mass transport analysis, adhesion force calculation, and the table of performance comparison of thin air gap solar-driven AGMD with previous reports (PDF)
