# Supplementary material for: Solar-Driven Thin Air Gap Membrane Distillation with a Slippery Condensing Surface
Source: Environ Sci Technol. 2024 Nov 16;58(47):21077–85. doi: 10.1021/acs.est.4c06470 (PMC11603763; doi:10.1021/acs.est.4c06470)
Supplement: Supplementary file 1 — es4c06470_si_001.pdf [file es4c06470_si_001.pdf]

Supporting Information

Solar-driven Thin Air Gap Membrane Distillation with Slippery Condensing Surface

Hongxia Li,[1,2] *Aikifa Raza,*[1] *Noora Ali AlMarzooqi,*[2,3] *Meera AlMehrzi,*[3,4] *Alaa Shaheen,*[3,4] *Faisal AlMarzooqi,*[3,4*] *TieJun Zhang*[1*]

[1] Department of Mechanical and Nuclear Engineering, Khalifa University of Science and Technology, P.O. Box 127788, Abu Dhabi, United Arab Emirates

[2] Technology Innovation Institute, P.O. Box 9639, Abu Dhabi, United Arab Emirates

[3] Department of Chemical and Petroleum Engineering, Khalifa University of Science and Technology, P.O. Box 127788, Abu Dhabi, United Arab Emirates

[4] Center for Membranes and Advanced Water Technology (CMAT), Khalifa University of Science and Technology, Abu Dhabi 127788, United Arab Emirates

[*]Address correspondence to: tiejun.zhang@ku.ac.ae; faisal.almarzooqi@ku.ac.ae

Summary of Supporting Information: 8 pages, 4 figures, 2 tables

Section S1: Mass Transport in AGMD Process
Table S1 Characteristics of PVDF membrane
Figure S1. (a) and (b) sensitivity analysis of permeate flux to condensation heat transfer coefficient and air gap thickness, respectively.
Section S2: Adhesion Forces from Solid Surface with Different CAHs
Figure S2. (a) Schematic of force balance analysis of droplet on the tilted surface. (b) and (c) Map of adhesion forces under different pairs of advancing and receding contact angles.
Section S3. Uncertainty Analysis of GOR
Figure S3. Calculation of GOR from experimental measures with tube heat loss correction.
Section S4. Heat Loss Analysis through Air Gap
Figure S4. Three different scenarios of water flooding in the air gap
Section S5. Comparison of Solar-driven AGMD Performance with Open Literature
Table S2. Comparison of solar-driven AGMD performance among the current work and previous reports

Section S1. Mass Transport in AGMD Process

The permeation flux J_{agmd} in the Air-gap Membrane Distillation (AGMD) process is determined by the following equation

$$J_{\text{agmd}} = \left(\frac{1}{B_{\text{m}}} + \frac{1}{B_{\text{ag}}}\right)^{-1} (P_{\text{fm}} - P_{\text{cf}}) \qquad \text{(S1)}$$

where B_m represents the mass transfer coefficient (MTC) of the membrane, and B_{ag} represents the one for air gap. P_{fm} is the partial vapor pressure at the interface between the feed and the membrane, and P_{cf} is the partial vapor pressure at the condensate film.

In the porous membrane, the mass transfer of water vapor is governed by Knudsen and molecular diffusion mechanisms. Accordingly, the membrane MTC is formulated as follows:

$$B_{\text{m}} = \left(\frac{1}{B_{\text{mol}}} + \frac{1}{B_{\text{kn}}}\right)^{-1} \qquad \text{(S2)}$$

where B_{mol} is the mass transfer coefficient of molecular diffusion and B_{kn} is the mass transfer coefficient of Knudsen diffusion across the membrane [S1][S2].

$$B_{\text{mol}} = \mathrm{M_w}/(\mathrm{P} - \mathrm{P_v}) \cdot \varepsilon_{\mathrm{m}}\mathrm{DP}/(\delta_{\mathrm{m}}\tau_{\mathrm{m}}\mathrm{RT}) \qquad \text{(S3)}$$

And,

$$B_{\text{kn}} = \frac{4d_p\varepsilon_m}{3\delta_m\tau_m}\sqrt{M_{\text{w}}/2\pi RT} \qquad \text{(S4)}$$

where ε_m, δ_m, τ_m, and d_p denote the porosity, thickness, tortuosity, and pore diameter of the membrane, respectively; R is the universal gas constant; P is the total pressure. D, the binary diffusion coefficient, is derived from the Fuller-Schettler-Giddings equation [S3][S4]:

$$D = 1.19 \times 10^{-4}\left(\frac{T_m^{1.75}}{P_m}\right) \qquad \text{(S5)}$$

with T_m as the mean temperature and P_m as the mean pressure of water vapor. The partial pressure of water vapor (P_v), exerted by pure water, is predicted by the Antoine equation[S4]:

$$P_v = exp\left(23.1964 - \frac{3816.44}{T - 46.13}\right) \qquad \text{(S6)}$$

Therefore, the vapor pressure at the feed and condensate film can be calculated at the corresponding temperature. The temperature of the condensate film on the condensing surface varies under different condensation heat transfer coefficients (HTC).

$$T_{cf} = T_c + \frac{J_{agmd}h_{lv}}{HTC} \qquad \text{(S7)}$$

where h_{lv} is the latent heat of the water, and T_c is the surface temperature of the condenser. By enhancing the condensation HTC, the T_{cf} can be reduced to have a lower vapor pressure near the condensate film and

a higher permeate flux. When the HTC increases from 10 to 80 kW m^{-2} K^{-1}, the permeate flux also increases. This increase in HTC is typically achieved through surface modification, facilitating the transition from film-wise condensation to dropwise condensation (refer to Figure S1a). Here, the flux values are normalized with the reference HTC of 40 kW m^{-2} K^{-1} under the feed temperature of 50 °C and coolant temperature of 20 °C.

In the feed stream, the effect of dissolved non-volatile solutes is accounted for by adjusting the water vapor partial pressure (P_{fm}) at the feed side liquid-vapor interface as follows:

$$P_{fm} = \gamma_w (1 - x_{fs}) P_v \quad \text{(S8)}$$

where γ_w is the activity coefficient of water, indicating the deviation from ideal mixture behavior, and x_{fs} is the molar fraction of the solute. The activity coefficient of an aqueous NaCl solution can be estimated as follows:

$$\gamma_w = 1 - 0.5x_{fs} - 10x_{fs}^2 \qquad \text{(S9)}$$

The partial vapor pressure at the feed side is calculated and verified by the seawater properties reported by MIT [S4]. With vapor transporting across the air gap, the MTC in the gap B_{ag} is calculated as follows, highlighting molecular diffusion as the primary mass transport mechanism for non-condensable gases within the air gap:

$$B_{ag} = \frac{M_w DP}{(P - P_v)\delta_{ag} RT_w} \qquad \text{(S10)}$$

where δ_{ag} is the air gap thickness. With the increase of air gap thickness, the MTC in the gap decreases, and thus the permeate flux decreases. Figure S1b shows the sensitivity of permeate flux to air gap width. The smaller the gap width is, the higher the flux is. The permeate flux is more sensitive to air gap width than the condensation HTC. It reveals that a thin air gap can enhance permeate flux if condensate droplets are effectively removed with a slippery surface.

Table S1 Characteristics of PVDF membrane

Manufacturer	Millipore[S5]
Nominal pore size	450 nm
Thickness	125 μm
Porosity	75%
Tortuosity	2.08
Material	Polyvinylidene fluoride (PVDF)

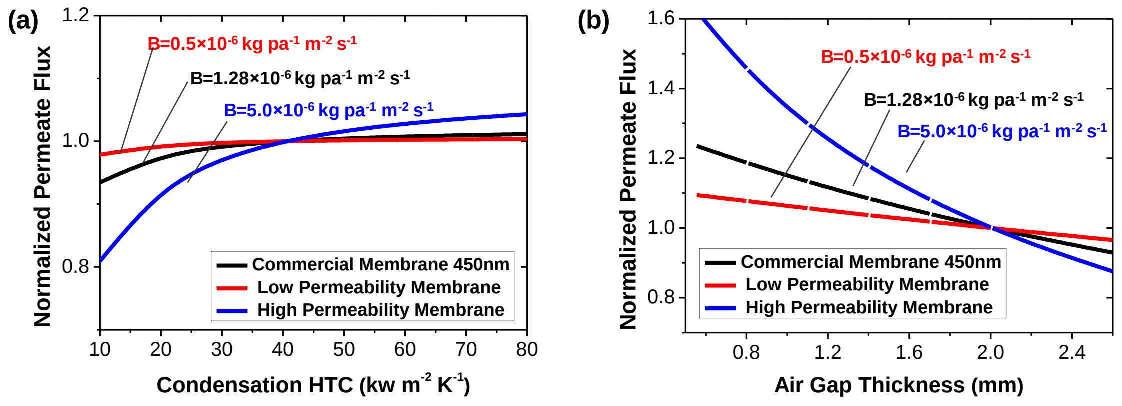

Figure S1. (a) and (b) sensitivity analysis of permeate flux to condensation heat transfer coefficient and air gap thickness, respectively.

Section S2. Adhesion Forces from Solid Surface with Different CAHs

When a droplet condenses on a solid surface, it experiences both the surface adhesion force and gravitational force. In the schematics of Figure S2, the contact area of the droplet is shaded in grey color, and the adhesion force is exerted along the contact line. The dynamic angle and the corresponding force values vary at different locations. The total adhesion force is then calculated by integrating the forces along the contact line. It is important to note that the contact line is no longer a perfect circle due to the droplet's deformation. In this study, with the following assumptions: (i) the contact line is approximated as a circle; (ii) the top half of the droplet exerts an adhesion force of $\gamma cos\theta_r$, while the bottom half exerts an adhesion force of $\gamma cos\theta_a$ (see the illustration in Figure S2a), we simplified the calculation of the net adhesion force as Eq. (S11):

$$F_{ad} = \pi R_b \gamma (cos\theta_r - cos\theta_a) \qquad \text{(S11)}$$

and

$$F_g = \rho V g sin\alpha \qquad \text{(S12)}$$

This gravitational force is balanced by surface adhesion forces at the equivalent state. The droplet volume V is calculated as:

$$V = \frac{\pi R_d^3}{3} \left(\frac{2-3\cos\theta + cos^3\theta}{sin\,\theta^3}\right) \qquad \text{(S13)}$$

where R_b is the base radius, R_d is the droplet radius, and $R_b = R_d sin\theta$. V is the droplet volume. γ is water surface tension, θ, θ_r, and θ_a are the static, receding and advancing contact angle, respectively. In the equation, α is the tilted surface angle and is 90 ° for the vertical surface. **Figure** S2 shows the adhesion force F_{ad} exerting on a 1 μm droplet under two different contact angles. Based on the contact angle hysteresis (CAH) measured for the three different surfaces, the adhesion force from the solid surface is marked in the color map. It can be found that the PDMS-coated quasi-liquid surface has the smallest adhesion to the droplet.

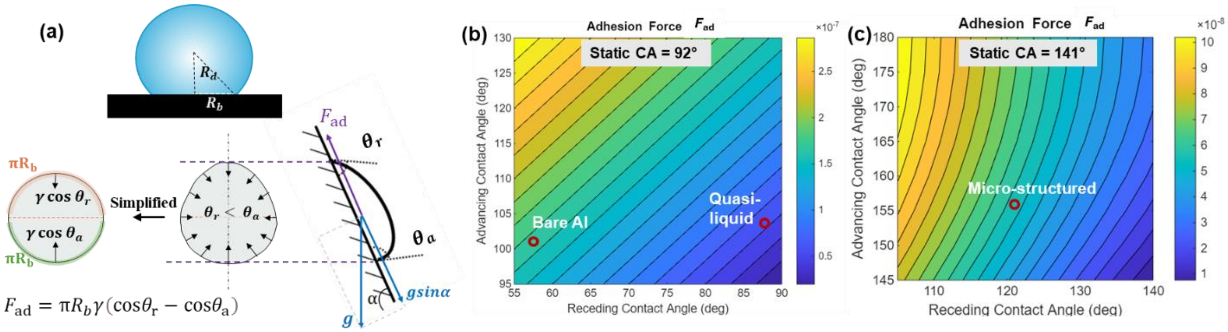

Figure S2. (a) Schematic of force balance analysis of droplet on the titled surface. (b) and (c) Map of adhesion forces under different pairs of advancing and receding contact angles.

Section S3. Uncertainty Analysis of GOR

This section provides the methodology used to perform uncertainty analysis and error propagation for the Gain Output Ratio (GOR) of the MD process. For conventional AGMD, it is the ratio of the evaporation enthalpy to the heat energy input from the feed water.

$$GOR = \frac{Q_{evap}}{Q_H} = \frac{\dot{m}h_{lv}}{V_f\rho c_p(T_{f,in}-T_{f,out})} \quad \text{(S14)}$$

Where $\dot{Q}_{\mathrm{evap}}$ is the evaporation enthalpy of water, and it is calculated as the production of permeate flux $\dot{m}_{\mathrm{p}}$ and latent heat h_{lv}. The total energy input is the sum of evaporation enthalpy, conduction loss from air gap, and other thermal loss. It can be obtained from the measured inlet temperature $T_{\mathrm{f,in}}$, outlet temperature $T_{\mathrm{f,out}}$, and the volume flow rate $\dot{V}_{\mathrm{f}}$ of the feed side.

Given the uncertainties associated with the measured values of permeate flux $u_{\dot{\mathrm{m}}}$, flow rate $u_{\mathrm{V_f}}$, inlet temperature $u_{\mathrm{T_{f,in}}}$, and outlet temperature $u_{\mathrm{T_{f,out}}}$, the uncertainty in GOR u_{GOR}, can be calculated using the propagation of uncertainty formula

$$u_{GOR} = \sqrt{\left(\frac{\partial GOR}{\partial \dot{m}}u_{\dot{m}}\right)^2 + \left(\frac{\partial GOR}{\partial V_f}u_{V_f}\right)^2 + \left(\frac{\partial GOR}{\partial T_{in}}u_{T_{f,in}}\right)^2 + \left(\frac{\partial GOR}{\partial T_{out}}u_{T_{f,out}}\right)^2} \quad \text{(S15)}$$

The partial derivatives of GOR with respect to each variable are:

$$\frac{\partial GOR}{\partial \dot{m}} = \frac{h_{lv}}{V_f\rho c_p(T_{f,in}-T_{f,out})} \quad \text{(S16)}$$

$$\frac{\partial GOR}{\partial V_f} = \frac{\dot{m}h_{lv}}{V_f^2\rho c_p(T_{f,in}-T_{f,out})} \quad \text{(S17)}$$

$$\frac{\partial GOR}{\partial T_{f,in}} = \frac{\dot{m}h_{lv}}{V_f\rho c_p\left(T_{f,in}-T_{f,out}\right)^2} \quad \text{(S18)}$$

$$\frac{\partial GOR}{\partial T_{f,out}} = \frac{\dot{m}h_{lv}}{V_f\rho c_p\left(T_{f,in}-T_{f,out}\right)^2} \quad \text{(S19)}$$

Substituting the above equations into Equation S15, we can obtain the overall uncertainty in the GOR value. For each measurement, the calculated the error of GOR are plotted accordingly in the Figures 5c, and 6c and 6d.

The value of GOR can be calculated from the measured water flux, inlet, and outlet temperature. Here is an example by using the measurement data in Figure 5b. Note that in Figure 5b, the temperature values are directly recorded from the thermal couples located near the inlet and outlet with around 10 cm tube distance. As highlighted in Figure S3a, the heat exchange between the tube and the ambient causes a fake temperature

difference ΔT_{tube}. To eliminate this, we conducted a set of calibration experiments by setting the same temperature between the feed and coolant side and using an impermeable membrane. In this way, there is no heat and mass transfer between the two sides. The measured temperature difference is only due to the heat exchange between the tube and the ambient. The value is plotted in Figure S3b. So when calculating the GOR, the $T_{f,out}-T_{f,in}$ is corrected by ΔT_{tube} from the directly measured data.

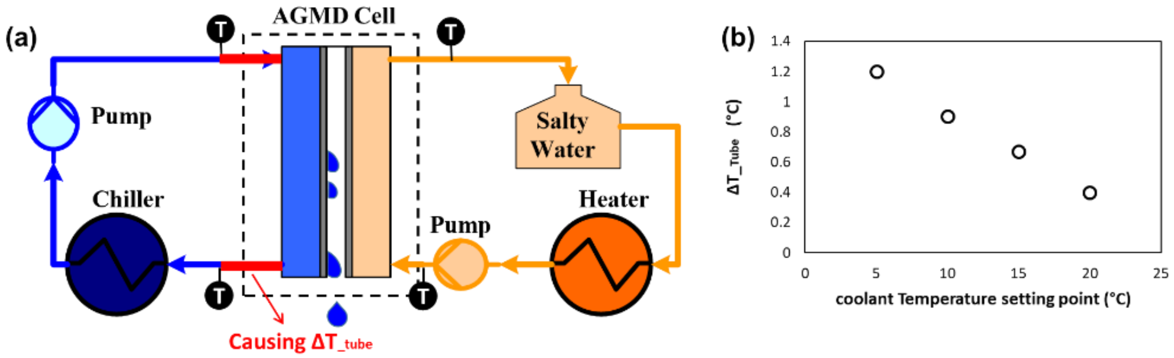

Figure S3. Calculation of GOR from experimental measures with tube heat loss correction.

Similarly, for solar-driven AGMD, the GOR is calculated as the ratio of evaporation enthalpy to the solar energy input.

$$GOR = \frac{Q_{evap}}{Q_H} = \frac{\dot{m}h_{lv}}{Aq_{solar}} \qquad \text{(S20)}$$

where A is the membrane surface area (4 cm × 8cm) under solar irradiance, q_{solar} is the solar irradiance (1000 w/m^2) during the experiments. With the same approach, the errors of GOR are calculated as below:

$$u_{GOR} = \sqrt{\left(\frac{h_{lv}}{Aq_{solar}}u_{\dot{m}}\right)^2 + \left(\frac{\dot{m}h_{lv}}{Aq_{solar}^2}u_{q_{solar}}\right)^2 + \left(\frac{\dot{m}h_{lv}}{A^2q_{solar}}u_A\right)^2} \qquad \text{(S21)}$$

Section S4. Heat Loss Analysis through Air Gap

In the heat loss analysis, the air gap was categorized into three scenarios: no flooding, partial flooding, and complete water flooding (see Figure S4).

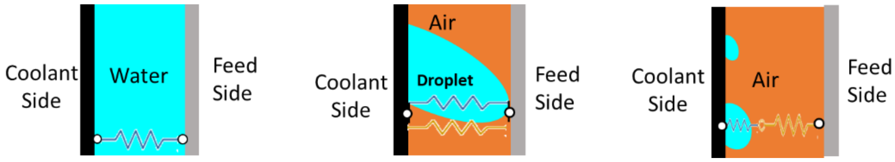

Figure S4. Three different scenarios of water flooding in the air gap

For the complete water flooding scenario, the thermal resistance is determined as

$$R_{WF} = \frac{\delta}{Ak_w} \qquad \text{(S22)}$$

where δ is the air gap thickness, A is the membrane surface area, and k_w is the thermal conductivity of water with the value of 0.59 W m^{-1}·K^{-1}. The R_{WF} is used to calculate the heat loss upper bound of the colour map in Figure 5d.

For the scenario of no flooding, the heat conduction occurs through the air and then the droplet, and thus the thermal resistance is calculated as the serial of the two resistances. Because of the variation of droplet sizes, the thermal resistance is the integration of all the droplet-air serials along the whole surface.

$$R_{NF} = \int_A (R_{droplet} + R_{air}) \qquad \text{(S23)}$$

The heat conduction resistance of a water droplet on the surface is a function of its contact angle θ and droplet radius r_d (also see the supporting information from our previous publication). It is calculated as

$$R_{droplet} = \frac{\theta}{4\pi r k_w sin\theta} \qquad \text{(S24)}$$

And the thermal resistance from air gap is

$$R_{air} = \frac{\delta - r_d}{\pi r^2 k_a} \qquad \text{(S25)}$$

However, it is challenging to have the accurate map of droplet sizes distribution on the surface. In particular, the sub-millimetre sized droplets have poor visibility, but they are majority in amount. After considering the poor conductivity of air k_a around 0.025 W $m^{-1} \cdot K^{-1}$ in comparison with water, we assume the resistance from water droplet is negligible. So, for the no flooding scenario, the thermal resistance is approximated as

$$R_{NF} \sim R_{air} = \frac{\delta - r_{d,avg}}{A k_a} \qquad \text{(S26)}$$

The R_{NF} is used to calculate the heat loss lower bound of the colour map in Figure 5d. This is the ideal AGMD configuration.

For the scenario of partial flooding, part of the membrane surface is directly water bridged to condensing surface, and part is still dry. So, the overall thermal resistance is the parallel of the two parts, calculated as:

$$R_{partial} = \left(\frac{1}{R_{water}} + \frac{1}{R_{air}}\right)^{-1} = \left(\frac{\delta}{A_w * k_w} + \frac{\delta}{A_a * k_a}\right)^{-1} \qquad \text{(S27)}$$

where A_w is water bridged area, A_a is the dry area, and the sum of A_w and A_a is the total membrane surface area A. In this work, the heat loss is calculated by subtracting the useful heat for water evaporation Q_{evap} from the total heat input Q_H, as shown below

$$Q_{loss} = Q_H - Q_{evap} = V_f \rho c_p \left(T_{f,in} - T_{f,out}\right) - \dot{m} h_{lv} \qquad \text{(S28)}$$

By allocating the Q_{loss} value in the colour map in Figure 5d, we estimated the flooding status in the air gap when using different condensing surfaces.

Section S5. Comparison of Solar-driven AGMD Performance with Open Literature

Table S2. Comparison of solar-driven AGMD performance among the current work and previous reports

This wok				
Materials	FLUX	GOR	Energy source	remarks
	LMH			
		C*0.01		
PVDF-ZrN	0.099	low		2 mm air gap with quasi-liquid condensing surface
PVDF-ZrN	0.145	low		1 mm air gap with quasi-liquid condensing surface
Solar-DCMD				
Materials	FLUX	GOR	Energy source	References
	LMH			
		C*0.01		
[MEM] MXene/PTFE	0.77	0.653	solar	ACS Appl. Mater. Interfaces 2022, 14, 4, 5265–5274
[MEM] Janus photothermal membrane	1.29	0.73	solar	ACS Appl. Mater. Interfaces 2021, 13, 26861–26869
[MEM] CB-PVA-PVDF	0.22	0.2145	solar	PNAS 2017, 114, 6936-6941
[MEM] CB-PVDF	1.5468	0.746	solar	J. Mater. Chem. A 5, 23712-23719
[MEM] rGO/pDA-PTFE	0.78	0.49	solar	Desalination 442, 1-7, 2018
[MEM] pDA-PVDF	0.538	0.45	solar	J. Mater. Chem. A 6, 18799–18807
[MEM] Fe3O4/PVDF/HFP	0.97	0.608	solar	Desalination 2020, 478, 114288
[MEM] cRSM-CNTs/PVDF	1.11	0.695	solar	Nano Energy 2019, 60, 567–575.
Solar-AGMD				
Materials	FLUX	GOR	Energy source	References
	LMH			
		C*0.01		
[MEM] PVDF 50AC	0.32	0.1604	solar	Chemical Engineering Journal 431 (2022) 133909
[MEM] PVDF-AC	0.31	0.21	solar	Desalination 541 (2022) 116031
[MEM] Commercial PVDF	0.47	0.3203	solar	Chemosphere 212 (2018) 554-562: nanofluids
[MEM] Dye-coated PTFE	0.885	0.605	solar	Water Research 127 (2017) 96-103
[MEM] TiN-NP PVDF	0.94	0.641	solar	Chemosphere 256 (2020) 127053

References

[S1] Im BG, Francis L, Santosh R, Kim WS, Ghaffour N, and Kim YD. “Comprehensive insights into performance of water gap and air gap membrane distillation modules using hollow fiber membranes,” *Desalination*, vol. 525, no. July 2021, p. 115497, 2022, doi: 10.1016/j.desal.2021.115497.

[S2] Noamani S, Niroomand S, Rastgar M, Azhdarzadeh M, and M. Sadrzadeh. “Modeling of Air-Gap Membrane Distillation and Comparative Study with Direct Contact Membrane Distillation,” *Ind. Eng. Chem. Res.*, vol. 59, no. 50, pp. 21930–21947, 2020, doi: 10.1021/acs.iecr.0c04464.

[S3] Ma XH, Zhou XD, Lan Z, Li YM, and Zhang Y. “Condensation heat transfer enhancement in the presence of non-condensable gas using the interfacial effect of dropwise condensation,” *Int. J. Heat Mass Transf.*, vol. 51, no. 7–8, pp. 1728–1737, 2008, doi: 10.1016/j.ijheatmasstransfer.2007.07.021.

[S4] https://web.mit.edu/seawater/
